# Application of a One-Health Approach for Dermatophyte Infections

**DOI:** 10.3390/tropicalmed11010016

**Published:** 2026-01-06

**Authors:** Deborah Cruciani, Manuela Papini, Sara Spina, Carla Sebastiani, Vincenzo Piscioneri, Alessandro Fiorucci, Silvia Crotti

**Affiliations:** 1 Istituto Zooprofilattico Sperimentale dell’Umbria e delle Marche “Togo Rosati”, Via G. Salvemini 1, 06126 Perugia, Italy; 2 Clinica Dermatologica di Terni, Dipartimento di Medicina e Chirurgia, Università degli Studi di Perugia, 06123 Perugia, Italy; 3 Nuovo Ospedale San Giovanni Battista, USL Umbria 2, 06034 Foligno, Italy

**Keywords:** *Canis lupus familiaris*, dermatomycoses, *Felis catus*, *Homo sapiens*, Italy, zoonosis

## Abstract

Dermatomycoses pose significant zoonotic and public health challenges, involving interactions among fungal agents, host immunity, and environmental reservoirs. Eight cases of dermatophyte infection involving five humans, two cats and one dog were investigated in the Umbria region applying a One-Health approach, as recommended by the CDC. Fungal isolates were identified by mycological and molecular methods as *Microsporum canis* (n = 4), *Nannizzia gypsea* (n = 3), and *Trichophyton mentagrophytes* var. *mentagrophytes* genotype III* (n = 1). The source of infection was identified in four cases enabling the implementation of appropriate treatment, removal of fomites, and environmental sanitization; as a result, no recurrences were observed. In the remaining cases, environmental assessments showed no fungal burden, indicating likely incidental transmission. Close cohabitation or contact with cats emerged as a risk factor. The patient’s medical history should always include exposure to animals in order to facilitate early recognition, correct management, and prevention. Interdisciplinary collaboration among dermatologists, veterinarians, and laboratory technicians is essential to optimize therapeutic outcomes and to prevent potential antifungal resistance phenomena. Moreover, continuous surveillance under a One-Health framework will enable better epidemiological understanding of dermatophyte species dynamics, particularly zoonotic agents.

## 1. Introduction

Dermatomycoses are superficial fungal infections affecting humans and animals. Dermatophytes are the keratinophilic and keratinolytic causative agents of the disease and dermatomycosis is one of the most common zoonotic diseases. According to their natural reservoir, dermatophytes can be classified as anthropophilic, zoophilic, and geophilic species, primarily affecting humans, animals, and soil, respectively [1,2]. It was hypothesized that this group of pathogens was originally geophilic and have gradually adapted to infect vertebrates over time through soil contact and carriage on animal fur [3]. Transmission occurs by direct or indirect contact and through contaminated fomites [4]. In recent decades, conventional pets, primarily dogs and cats, have assumed increasing importance in domestic settings and could facilitate the contagion. Globally, a total of 37 regions reported human dermatophyte infections linked to animal contact, especially among the European continent [5]. Additionally, it is known that fungal spores can survive in the environment for years under optimal temperature and humidity conditions [4]. Generally, children and young animals are more predisposed to dermatomycosis as well as other vulnerable subjects having an impaired immune system (e.g., HIV, cancer, pregnancy, etc.) [4,6].

In humans, dermatomycoses can be clinically classified by the infection site (e.g., *tinea corporis*, *tinea capitis*, *tinea cruris*, etc.) whereas in animals they are commonly referred to as ringworm or *tinea* [1]. Both in humans and animals, clinical signs include any combination of hair loss, scaling, crusting, erythema, papules, hyperpigmentation, and variable pruritus [5,7]. The location of lesions can suggest how the disease has been transmitted: for example, in hunting dogs lesions first develop on the head and legs or paws or digits [8]. In cats, alopecic and inflamed lesions are uncommon [7]; they often develop on the face, ears, and muzzle and then progress to the paws and tail, whenever evident [8]. According to the established literature, the most common zoonotic dermatophytes include *Microsporum canis*, *Nannizzia gypsea*, *Trichophyton mentagrophytes* complex, *T. verrucosum*, and other *Trichophyton* spp. [5].

Therefore, pathogenesis involves complex interplay between the fungal agent, host defense, and the environment [9]. Moreover, climate change and the rapid emergence of drug-resistant species are producing epidemiological shifts. Particularly, high humidity and warm environmental temperature increase the incidence of dermatomycoses, especially during spring and summer in tropical and subtropical countries like India where *T. mentagrophytes* complex is now more frequently observed than *T. rubrum* [4,9,10].

Starting from the early 2000s, investigations into dermatophyte cases have also been extended to settings involving close contact with patients and fungal infection management has become an important public health issue. In particular, in the last years, the U.S. Centers for Disease Control and Prevention (CDC) has advocated for the One-Health framework in managing fungal diseases, which considers environmental and animal factors in examining the spread and resistance development of human diseases [11]. In detail, when a patient is infected with zoophilic dermatophytes, it is advisable to form a collaborative framework between dermatologists and veterinarians to identify the animal source and prevent further spread or re-infection. The removal of the source of infection (SOI) contributes not only to human and animal health, but also to the reduction in the use of drugs, an aspect that is not negligible, to contrast the growing antifungal resistance phenomena. Otherwise, if the SOI is not investigated, any reliable hypothesis about it could be supposed, with the risk of maintaining a high load of spores within the domestic environment.

A One-Health approach was previously adopted by the authors to menage a presumptive zoonotic Kerion in a child, giving successful results [12]. The aim of this study was to apply the same approach in the other eight cases and improve it involving not only humans but also animals as index patients.

## 2. Materials and Methods

This study compiles eight cases of dermatomycosis from 2018 onwards. It involved five humans (*Homo sapiens*, Linnaeus, 1758), two cats (*Felis catus*, Linnaeus, 1758), and one dog (*Canis lupus familiaris*, Linnaeus, 1758) as index patients. All of them lived in Umbria region (Central Italy) and their medical history is described hereafter.

**Figure 1:**
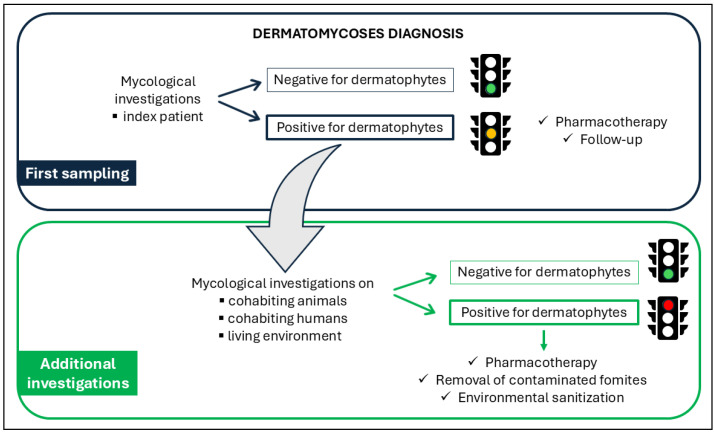
Schematic workflow of the One-Health approach implemented in this study, illustrating the interactions among human, animal, and environmental health components.

The One-Health approach adopted to menage these fungal infections is showed in Figure 1.

In detail, the procedure consisted of two sampling steps. The first sampling was performed during the index patient clinical examination to reach the diagnosis: since a dermatomycosis occurred, a second sampling became essential to investigate the environment where the index patient lived as well as other humans or animals living in the same context. This healthcare framework was designed to reveal the SOI in order to eliminate it and reduce the risk of potential recurrences.

### 2.1. Study Cases

#### 2.1.1. Case 1 (October 2018)

A 40-year-old single man presented with a mildly pruritic, minimally inflamed lesion on his arm consistent with *tinea corporis* (ringworm). He declared close contact with an adult cat and a recently adopted stray kitten. Neither animal exhibited any visible lesions.

#### 2.1.2. Case 2 (November 2020)

A 28-year-old single woman showed a lesion attributable to *tinea manuum*. She volunteered at a wildlife rehabilitation center, where she frequently handled several animals, including red foxes and rabbits, for clinical care. She was also the owner of a dog that did not exhibit dermatological lesions.

#### 2.1.3. Case 3 (December 2020)

A 10-year-old girl (patient A) showed a Kerion Celsi lesion represented by a crusted, purulent mass surrounded by erythematous alopecic areas. Her sister (patient B), aged 8, exhibited *tinea faciei*, with lesions approximately 1 cm in diameter on both cheeks. The parents reported that the family lived in a house with free access to open spaces and had daily close interactions with three adult cats and two kittens. Neither the parents nor the animals showed any visible lesions.

#### 2.1.4. Case 4 (June 2021)

A 40-year-old woman showed an inflamed and pruritic lesion on the forearm consistent with *tinea corporis* (ringworm). She lived with her partner together with three hunting dogs with whom she had a close relationship, regularly engaging in agility training and conformation competitions. The partner did not show dermatological lesions, and the dogs were in health and regularly vaccinated.

#### 2.1.5. Case 5 (May 2023)

A 6-year-old girl exhibited *tinea corporis*, characterized by mildly inflamed, pruritic lesions approximately 1.5 cm in diameter on both arms. Her medical history included cohabitation with parents, a sister, one kitten, two adult cats, three dogs, and one rabbit. None of the family members nor the animals exhibited clinical lesions attributable to dermatomycosis.

#### 2.1.6. Case 6 (January 2022)

The index patient was a kitten living in a stray cat colony and subjected to a complete health check-up prior to being adopted by a new family. Even if mycological testing was performed, the adoption was finalized before the diagnostic results were available, as the kitten showed no dermatologic lesions at the time. Unfortunately, once the culture test returned positive, dermatomycosis-related lesions appeared in household members.

#### 2.1.7. Case 7 (August 2022)

A 6-month-old kitten presented with an alopecic lesion on the tail. It had recently been adopted by a new family in which two asymptomatic adult cats also resided. In the new house the animals had access to a terrace on the fourth floor of a building, with no access to gardens or green areas.

#### 2.1.8. Case 8 (November 2023)

In a 5-year-old dog an alopecic patch with a low degree of inflammation was observed on the nasal *planum*. No other lesions were detected. The dog had free access to the garden but did not engage in hunting. The only cohabitant was the owner; no other animals or humans shared the household.

### 2.2. First Sampling

In humans, the first sampling was performed by a dermatologist during clinical examination at the Dermatology Unit of Santa Maria Hospital in Terni (Umbria region). Five skin scrapings were collected using a scalpel, from the anatomic site where the dermatological lesion was present (as further specified in Table 1). In case 3A the sample consisted of hairs plucked with tweezers from the area surrounding the Kerion lesion.

Animal samples were collected by a veterinarian and conferred to Istituto Zooprofilattico Sperimentale dell’Umbria e delle Marche “Togo Rosati” (IZSUM, Perugia, Umbria region). In the asymptomatic kitten (case 6), sampling was performed using the MacKenzie modified technique by employing a scalp brush over the entire body surface [13]. In symptomatic animals (cases 7 and 8), hair surrounding the ringworm lesions on the tail and nasal *planum* was sampled using sterile tweezers.

### 2.3. Mycological and Molecular Investigations

The samples were inoculated on Dermasel Agar, incubated at 25 ± 1 °C in aerobiosis, and daily monitored. Fungal growth was observed in all cases within 7 days. The colonies exhibited macroscopic and microscopic features typical of dermatophytes. PCR and DNA sequencing were performed to identify the fungal species. DNA was extracted using QIAamp DNA mini kit (QIAGEN^®^, Valencia, CA, USA) following a modified Gram-positive protocol (Appendix D: Protocols for Bacteria, Isolation of genomic DNA from Gram-positive bacteria) and subjected to hemi-nested PCR using DMTF18SF1 (5′-CCAGGGAGGTTGGAAACGACCG-3′) as the common forward primer and DMTF28SR1 (5′-CTACAAATTACAACTCGGACCC-3′) and DMTFITS1R (5′-CCGGAACCAAGAGATCCGTTGTTG-3′) as the reverse primers for the first and second step, respectively [14,15]. Electrophoresis on 2% agarose gel stained with Midori Green Advance (NIPPON Genetics^®^, Düren, Germany) showed amplicons of about 400 bp. They were purified by QIAquick PCR Purification Kit (QIAGEN^®^, Valencia, CA, USA) and then subjected to Sanger sequencing using BrilliantDye Teminator Cycle Sequencing Kit (Resnova^®^, Waltham, MA, USA) and 3500 Genetic Analyzer (Applied Biosystems^®^, Foster City, CA, USA). Consensus sequences were created by BioEdit Sequence Alignment Editor software version 7.2.5 [16] and then aligned in the Westerdijk Fungal Biodiversity Institute database [17]. Dermatophyte species identification through direct alignment was considered reliable except for differentiating species within the *T. mentagrophytes* complex. In this case, another PCR assay using primers ITS1 and ITS4 [18] was more recently performed. The amplicon obtained of about 700 bp was sequenced following the procedure described above. In detail, to distinguish *T. mentagrophytes* var. *mentagrophytes* from *T. mentagrophytes* var. *interdigitale*, a single nucleotide polymorphism at position 94 was evaluated [19]. Moreover, the strain obtained was assigned to a specific genotype, based on the classification schemes described by Nenoff et al. [20] and Taghipour et al. [21].

All the sequences obtained from the first sampling have been subsequently deposited in the GenBank database under the accession number listed in Table 1.

### 2.4. Second Sampling (Additional Investigations)

Based on the One-Health framework (Figure 1), additional investigations were carried out after the diagnosis of dermatomycosis. In detail, if the infection raised on a human patient, other humans or animals living in the same context were investigated. Similarly, humans or other animals were examined if the infection raised on an animal. In both scenarios, the environment where the patients usually lived or worked was also investigated, particularly pieces of furniture (e.g., sofas, beds) and various objects (e.g., carpets, cats’ and dogs’ beds). Fourteen human and twenty animal samples were represented by hair collected through the MacKenzie modified technique [13] as described above, whereas forty-one environmental samples were harvested through surface swabs. All samples were then submitted to the same analytical process used to analyze the specimens of the first sampling.

### 2.5. Phylogenetic Analysis

In order to correlate the strains isolated from human patients, animals, and the environment, a bioinformatic analysis was performed and the Neighbor-Joining method was used to construct a phylogenetic tree. This analysis was applied exclusively to *M. canis* strains, which were the only ones recovered during the second sampling phase across all three potential sources of infection (SOI).

## 3. Results

The results of the first and the second sampling are shown in Table 1.

**Table 1:** Human and animal clinical cases: details of clinical lesions and results of the first and the second sampling. N/A = not applicable, source to be sampled not present in the clinical case investigated; * = concurrently lesion appearance with the positive result of the cat’s culture test; +: positive; −: negative; /: not performed.

		First Sampling		Second Sampling			Results	
Case	Index Patient	Clinical Lesion	Fungal Species (Accession Number)	Cohabiting Animals	Cohabiting Humans	Living Environment	Mycological Examinations	Molecular Analyses
1	Human	*Tinea corporis* (arm)	*M. canis* (OR095643)	**Cat** **Kitten**	N/A	**Cat’s bed** **Carpet** Sofa Bed	**+** **+** / **+** **+** − −	*M. canis* (PX692089) *M. canis* (PX692090) */* *M. canis* (PX692091) *M. canis* (PX692092) / /
2	Human	*Tinea manuum* (hand)	*T. mentagrophytes* var. *mentagrophytes* genotype III* (OR095660)	Dog	N/A	Dog’s bed Carpet Sofa Bed	− / − − − −	/ / / / / /
3	Human	Kerion Celsi (scalp) (A) *Tinea faciei* (cheek) (B)	*M. canis* (A, B) (OR095728)	**Cats (n = 3)** **Kitten (n = 2)**	Parents	**Cats’ beds (n = 3)** Carpet Sofa Bed	**+** **+** − **+** − − −	*M. canis* (PX692080, PX692081, PX692082) *M. canis* (PX692083, PX692084) / *M. canis* (PX692085, PX692086, PX692087) / / /
4	Human	*Tinea corporis* (forearm)	*N. gypsea* (OR095664)	Dogs (n = 3)	Partner	Dogs’ beds (n = 3) Carpet Sofa Bed	− − − − − −	/ / / / / /
5	Human	*Tinea corporis* (arms)	*M. canis* (OR095729)	**Kitten** Cats (n = 2) Dogs (n = 3) Rabbit	Parents Sister	Cats’ beds (n = 2) Dogs’ beds (n = 3) Carpet Sofa Bed	**+** − − − − − − − − − −	*M. canis* (PX692088) / / / / / / / / / /
6	Kitten	No visible lesions	*M. canis* (OR095726)	N/A	**Owners (n = 3)** *	**Cat’s bed** **Carpet** **Sofa** **Bed**	− **+** **+** **+** **+** **+**	/ *M. canis* (PX692093, PX692094, PX692095) *M. canis* (PX692096) *M. canis* (PX692097) *M. canis* (PX692098) *M. canis* (PX692099)
7	Kitten	Ringworm (tail)	*N. gypsea* (OR095725)	Cat (n = 2)	Owners (n = 4)	Cats’ beds (n = 2) Carpet Sofa Bed	− − − − − −	/ / / / / /
8	Dog	Ringworm (nasal *planum*)	*N. gypsea* (PX574360)	N/A	Owner	Dog’s bed Carpet Sofa Bed	− − − − − −	/ / / / / /

**Figure 2:**
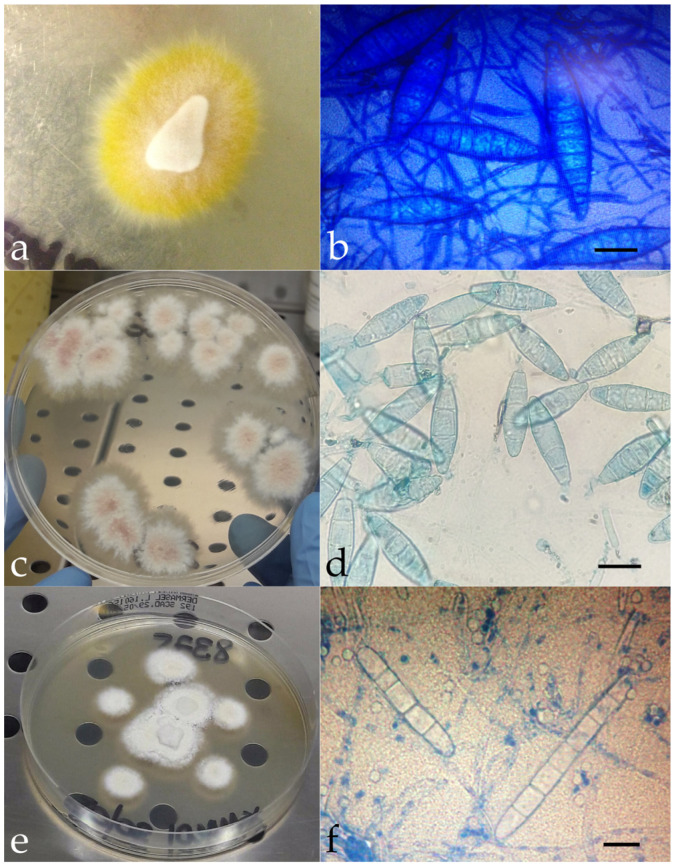
Macroscopic appearance of the isolated colonies referable to *M. canis* (**a**), *N. gypsea* (**c**), and *Trichophyton* spp. (**e**). Microscopic features of *M. canis* ((**b**), methylene blue staining, ×100, bar 20 μm), *N. gypsea* ((**d**), methylene blue staining, ×100, bar 10 μm), and *Trichophyton* spp. ((**f**), methylene blue staining, ×100, bar 10 μm). Molecular investigations identified three different fungal species as the causative agent of dermatomycosis: *Microsporum canis* (n = 4, cases 1, 3, 5, and 6), *Nannizzia gypsea* (n = 3, cases 4, 7, and 8), and *Trichophyton mentagrophytes* var. *mentagrophytes* genotype III* (n = 1, case 2). The SOI was identified in some instances; in others, it remained hypothetical.

Mycological investigations conducted on human, animal, and environmental samples showed flat, powdery colonies with ragged margins and a lemon-yellow reverse in cases 1, 3, 5, and 6 (Figure 2a). Colonies with similar macroscopic features and a wine-colored reverse were obtained in cases 4, 7, and 8 (Figure 2c). Microscopically, these colonies showed fusiform and multiseptate macroconidia (Figure 2b,d). In case 2, the colonies appeared whitish with smoother margins and a more cottony texture (Figure 2e); cigar-shaped structures were visible microscopically, suggesting *Trichophyton* as the likely genus (Figure 2f).

### 3.1. Human Cases

*Microsporum canis* was identified as the causative dermatomycosis agent in three human cases (1,3, and 5). In cases 1 and 3 the same strain was also recovered on the adult cats and kittens and the environment. At the second sampling, in case 1, the positivity rates in animals and in the environment were 10% (n = 2/20) and 7.31% (n = 3/41), respectively, whereas in case 3, from 25% of the animals (n = 5/20) and 4.87% of the environmental samples (n = 1/41), the same dermatophyte isolated in the first sampling was identified. In case 5, *M. canis* was subsequently observed in the kitten (5%, n = 1/20).

*Trichophyton mentagrophytes* var. *mentagrophytes* genotype III* and *Nannizzia gypsea* were diagnosed in cases 2 and 4, respectively. In both cases cohabiting animals and the environment tested negative for dermatophytes.

The family members living with the index human patients of cases 3, 4, and 5 tested negative on the second sampling.

### 3.2. Animal Cases

*Microsporum canis* was identified as the causative dermatomycosis agent in case 6. The same strain was also detected in the three owners, who were the only human subjects positive at the second sampling of this investigation, with a percentage of 21.43% (n = 3/14). In addition, the cat bed, carpet, sofa, and bed of the adoptive home also tested positive for *M. canis*, with a percentage of 9.75% (n = 4/41).

In cases 7 and 8, *N. gypsea* was isolated from the first sampling performed on the kitten and the dog, respectively. In case 7, no other animals nor family members nor the environment were infected or contaminated as well as the dog’s owner and the environment in case 8.

### 3.3. Phylogenetic Analysis Results

**Figure 3:**
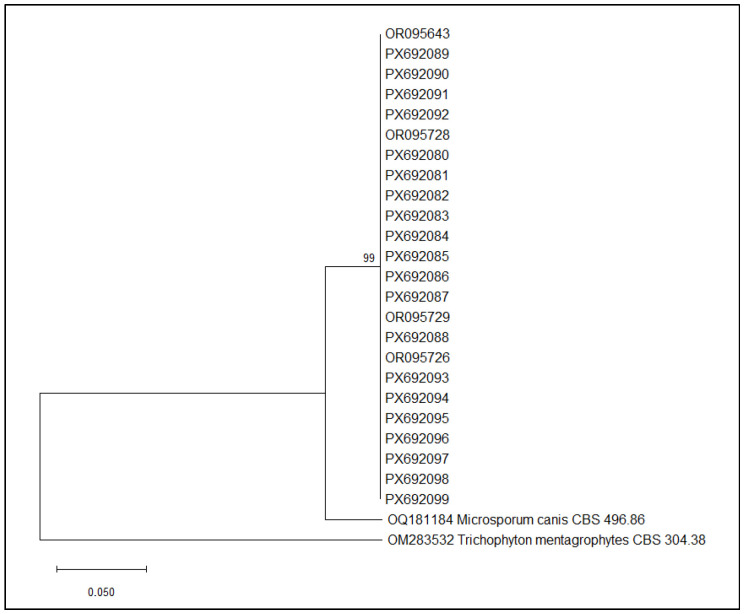
Neighbor-Joining phylogenetic tree of *M. canis* sequences obtained in cases 1, 3, 5, and 6. Maximum Composite Likelihood method, 1000 bootstraps (MEGA12 software, version 12.0.11).

The phylogenetic analysis included exclusively *M. canis* strains isolated from human patients, animals, and the environment in cases 1, 3, 5, and 6. The phylogenetic tree obtained is shown in Figure 3.

The phylogenetic analysis revealed a well-supported monophyletic cluster comprising all *M. canis* isolates included in this study. Bootstrap support at the major internal node is high (99%), indicating strong confidence in the branching pattern and suggesting that these isolates share a recent common ancestry. Within this clade, the sequences show minimal divergence, forming a compact group similar to *M. canis* CBS 496.86 and clearly separated from the outgroup *T. mentagrophytes* CBS 304.38.

## 4. Discussion

A One-Health approach involving dermatologists, veterinarians, and laboratory technicians was adopted to collect, analyze, identify, and menage eight cases of dermatomycoses detected in five humans and three animals. This procedure was previously conducted by the same authors in a presumptive zoonotic Kerion case in a child [12]; however, in the present study it was applied both in case of human and animal index patients. It consisted of two steps: the first to identify the fungal agent and the second to reveal the most probable SOI considering the natural reservoir of the dermatophyte species.

The causative agent of dermatomycosis was identified in all cases combining conventional and molecular methods avoiding mistakes due to the macroscopic and microscopic appearance of the strains [22]. *Microsporum canis*, *Nannizzia gypsea*, and *Trichophyton mentagrophytes* var. *mentagrophytes* genotype III* were the species detected in this study.

As it concerns *M. canis*, *tinea capitis* is the most common infection caused by this species, characterized by erythematous and pruritic alopecic patches with crusts and scales [5]. In children, the infection may lead to the secondary development of Kerion Celsi, involving a deep follicular invasion, resulting in edematous and pustular lesions with alopecia [23], like in case 3A. *Tinea corporis*, observed in cases 1 and 5, is reported as another clinical presentation by Gupta et al. [5]. *Microsporum canis* is widespread worldwide, particularly in Europe, the eastern Mediterranean, and South America and plays an important zoonotic role [24]. It is classified among zoophilic species, most found in cats and dogs. Particularly, stray cats are the main source of human dermatomycoses in urban areas; they could be symptomatic or, more frequently, they could act as healthy carriers [25]. In this study, *M. canis* was detected in cases 1, 3, 5, and 6, all of which involved cats that were identified as the SOI. In particular, the medical history of case 6 highlights the importance of a complete health check-up prior to adopt animals, especially kittens, even if they do not show any clinical signs, to safeguard future cohabiting humans, other animals, and the environment.

*Nannizzia gypsea* was identified in cases 4, 7, and 8. In the literature, dogs represent the most commonly reported animal host for this geophilic species followed by cats [5]. It is hypothesized that animals initially contract *N. gypsea* infections through contact with soil which can then spread within household environments. Fortunately, in cases 7 and 8, second sampling did not give evidence of fungal contamination apart from the animals, probably thanks to an early diagnosis and good hygienic practices of their owners. However, the SOI may be hypothesized. In detail, in case 7 it was supposed that the SOI could have arisen from the stressful, high-density conditions, given that the kitten originated from a colony; probably, in case 8 the nasal *planum* lesion appeared since the dog usually smell the soil. Moreover, several studies reported potential zoonotic transmissions which predominantly involved a contact history with dogs or cats [5]. In case 4, the human patient declared a close relationship with her three hunting dogs but the animals, her partner, and the environment tested negative at the second sampling. Thus, the SOI was supposed to be as accidental; the infection was most likely acquired in an environment where the patient only patronizes occasionally (e.g., a gym, pool).

*Trichophyton mentagrophytes* var. *mentagrophytes* genotype III* identified in case 2 (Germany type I) is the most prevalent in Europe and it is primarily associated with zoonotic transmission, inducing moderate to severe inflammatory lesions [26]. It was isolated only in the index patient. Despite she handled wild animals and owning pets, the animals and the environment tested negative at the second sampling. Therefore, the SOI was supposed to be accidental, as in case 4.

In this study, the One-Health approach allowed to analyze a further 75 samples, in addition to the eight index patients, yielding positivity percentages of 21.43% (n = 3/14), 40% (n = 8/20), and 21.95% (n = 9/41) among cohabiting humans, cohabiting animals, and environmental samples, respectively. In particular, the positive results detected at the second sampling were all associated with *M. canis*. This underscores the critical importance of this approach to uncover the SOI and to elucidate the interconnections between humans, animals, and their shared environment. Considering the medical history of the human index patients analyzed in this study and in related research, *M. canis* emerged as the predominant infectious agent, especially in kittens and children—underscoring that close contact with pets can facilitate dermatophyte transmission and thus represents a significant risk factor [24]. Moreover, overcrowded environments, such as kennels or cat colonies, constitute critical situations where regular monitoring of major infectious diseases is advisable. This becomes particularly crucial at the time of adoption, when animals are transferred into new human and environmental contexts, to safeguard health and safety. Another authors’ consideration was that phylogenetic clustering strongly suggested that within each case the environmental, animal- and human-derived isolates were highly genetically similar, pointing to a common source of contamination or transmission within the household. These results supported the hypothesis of household-specific transmission or environmental contamination of *M. canis*. Considering that it is a zoophilic species and that its transmission occurs through direct contact or via spores present in the environment, it was plausible to hypothesize that the primary source of infection was represented by the animals.

Thanks to the SOI identification, an appropriate pharmacological treatment of patients was prescribed, the contaminated objects were removed, the environment was sanitized, and no further recurrences were observed. When the source of infection was not identified, the One-Health investigation was still useful in conclusively excluding potential sources—since they tested negative—and in confirming that the patient’s environment could be considered healthy.

A highly ambitious goal for the future is to raise awareness in the scientific community about the importance of adopting this protocol—most critically in recurring cases—by integrating data on animal contact or cohabitation into the medical history. Otherwise, the problem is unlikely to be adequately addressed.

## 5. Conclusions

Although the proposed One-Health approach had already been adopted by other authors, the use of molecular techniques and the systematic sampling of all three possible dermatomycoses SOI—human, animal, environment—allowed more precise epidemiological information to be obtained, especially in view of the ongoing taxonomic evolution of dermatophytes.

Through this work experience, the authors hope that this operating procedure—already advocated by the CDC—can become part of routine diagnostic practice for dermatomycoses rather than remaining limited to pilot or occasional studies.

## Data Availability

The original data presented in the study are openly available in GenBank database.

## Abbreviations

The following abbreviations are used in this manuscript:

CDC	U.S. Centers for Disease Control and Prevention
IZSUM	Istituto Zooprofilattico Sperimentale dell’Umbria e delle Marche “Togo Rosati”
PCR	Polymerase chain reaction
SOI	Source of infection
